# Anaesthetic management and incidence of anaesthetic complications in dogs undergoing balloon valvuloplasty for treatment of pulmonic stenosis: a retrospective study

**DOI:** 10.3389/fvets.2025.1595738

**Published:** 2025-08-22

**Authors:** Lydia Hjalmarsson, Cristina Bianchi, Joshua Hannabuss, Thaleia Stathopoulou

**Affiliations:** Royal Veterinary College (RVC), London, United Kingdom

**Keywords:** inhalant anaesthesia, balloon valvuloplasty, blood pressure, dogs, intravenous anaesthesia, propofol

## Abstract

A retrospective analysis of dogs undergoing balloon valvuloplasty of the pulmonic valve between April 2014 and March 2023 was performed. Anaesthetic records from 44 dogs were included in the analysis. Dogs were grouped according to anaesthetic maintenance agent used, inhalational agent with partial intravenous anaesthesia (PIVA, *n* = 31) or propofol total intravenous anaesthesia (TIVA, *n* = 13). Variables including invasive blood pressure, heart rate, incidence of arrhythmias and requirement for interventions in the form of fluid bolus, anticholinergics and vasopressors were compared. Incidence of hypotension was significantly lower in dogs maintained on TIVA (40%) compared to PIVA (79%) (*p* = 0.008). Total duration of hypotension was shorter in dogs maintained on TIVA (median 40, interquartile range (IQR) 10–62.5 *versus* median 65, IQR 17.5–110 min) (*p* = 0.003). Systolic, mean and diastolic blood pressures were significantly higher in patients maintained on TIVA (107 ± 18, 73 ± 10 and 59 ± 8 mmHg respectively) compared to those maintained on PIVA (96 ± 15, 65 ± 9 and 52 ± 8 mmHg respectively, *p* = 0.039, *p* = 0.0079, *p* = 0.0156). No significant differences in incidence of arrhythmias (*p* = 0.292) and heart rate (88 *±* 14 and 88 *±* 18 beats minute^−1^ respectively) (*p* = 0.953) were seen between the two groups. There was also no significant difference in the number of interventions required to maintain normotension (*p* = > 0.1).

## Introduction

Pulmonic stenosis represents 31–32% of diagnosed congenital heart disease cases in dogs (1, 2), with an increased prevalence in brachycephalic breeds (3) and male dogs (1). Pulmonic stenosis can be classified as subvalvular, valvular or supravalvular based on the localisation of the lesion, with valvular stenosis being the most common form (4).

Significant pulmonic valve stenosis can be associated with symptoms such as exercise intolerance, syncope, congestive heart failure and sudden death (4, 5). Patients with mild peak transvalvular pulmonary gradients can often remain asymptomatic throughout life (5). Pulmonic balloon valvuloplasty has been demonstrated to alleviate clinical signs and prolong survival in dogs with severe pulmonic stenosis (4–6). Balloon valvuloplasty is a minimally invasive intervention (accessed typically through the femoral or jugular vein) which uses a percutaneous transluminal angioplasty balloon catheter to dilate the stenotic lesion (7). Despite undergoing balloon valvuloplasty, the valve is not returned to normal function and stenosis can reoccur (3). However, beneficial effects have been shown to be maintained a year after balloon valvuloplasty (8), with symptoms eliminated in 80% of dogs with long term post operative follow up (9).

In small animal veterinary practice, inhalation anaesthesia is commonly used for maintenance of general anaesthesia (10) with licensed formulations of both isoflurane and sevoflurane available. Isoflurane is a widely used inhalation anaesthetic in small animal veterinary practice, providing a stable depth of anaesthesia (11). However, its use can be accompanied with dose dependent cardiopulmonary depression (12). Sevoflurane has no clinically significant differences in heart rate, blood pressures or respiratory rate when compared to isoflurane in dogs (13). Partial intravenous anaesthesia (PIVA) refers to the infusion of intravenous drugs alongside inhalation anaesthesia (14). Using PIVA facilitates the use of reduced inhaled concentrations of volatile agents which can provide superior haemodynamic stability and provides a good quality of anaesthesia and recovery (14). Total intravenous anaesthesia (TIVA) refers to the maintenance of general anaesthesia by intravenous infusion only (15). Propofol is an intravenous induction agent that acts at GABAA receptors (16) and is the anaesthetic agent that is most commonly used for TIVA in dogs (17). While propofol TIVA provides advantages such as reduced operating room pollution, easy titration and smooth anaesthetic recovery (18), it can be also associated with dose-dependent cardiorespiratory depression (10).

General anaesthesia for balloon valvuloplasty presents several challenges due to the underlying cardiac disease of these patients and the side effects related with the technique itself. Ramos et al. (7) reported the most common anaesthetic complications in dogs undergoing balloon valvuloplasty to be cardiac arrhythmias (53.8%), hypotension (48.7%), bradycardia (20.5%) and desaturation of haemoglobin (17.9%). Arrhythmias during catheterisation of the right ventricular outflow tract have been reported in up to 87% of dogs (19). Patient mortality in the perioperative period has been reported in 2.6–6% of cases with reported causes of death including rupture of the pulmonary artery, hypotension followed by arrest, euthanasia secondary to chylothorax and suspected pulmonary thromboembolism (7, 19). Currently, no consensus exists on the optimal anaesthetic protocol to manage veterinary patients undergoing balloon valvuloplasty. While veterinary studies describe the use of inhalant anaesthetics in these patients (19, 20), to the author’s knowledge, only one case report documents the use of total intravenous anaesthesia (TIVA) in veterinary patients undergoing balloon valvuloplasty (21).

The use of both PIVA and TIVA have been assessed in human literature in patients undergoing anaesthesia for cardiac interventions. Despite the suggested cardioprotective effects of volatile anaesthetics (22), a systematic review and meta-analysis carried out by Beverstock et al. (23) found no significant differences in mortality or cardiac marker levels when comparing volatile anaesthesia with TIVA. Another review by Makkad et al. (24) concluded that the evidence comparing the use of volatile anaesthetics and TIVA in cardiac surgery was inconclusive, indicating that either technique can be considered an appropriate option. In veterinary medicine, TIVA using propofol alone has been associated with improved mean arterial pressures (10, 25, 26), higher systemic vascular resistance, higher heart rates (26) and better preservation of aortic pressures (27) than isoflurane anaesthesia.

This study aims to compare the incidence of anaesthetic complications and requirement for interventions to maintain cardiovascular stability with PIVA and total intravenous anaesthesia using a propofol infusion in dogs undergoing balloon valvuloplasty for pulmonic stenosis. We hypothesized that there would be no significant difference in the number of cardiovascular complications or the number of required interventions in patients undergoing balloon valvuloplasty when anaesthesia was maintained with PIVA or TIVA.

## Materials and methods

### Animals

An electronic review of the operating theatre record database of the Queen Mother Hospital for Animals identified dogs that had undergone balloon valvuloplasty between April 2014 and March 2023. Medical records including echocardiographic findings and anaesthetic records were obtained for each patient. Dogs were included in the study if they were anaesthetised for a balloon valvuloplasty for pulmonic stenosis during this period. Dogs were excluded if anaesthetic records were not available. Ethical approval was not required for this study due to the retrospective nature.

### Data collection

For each animal, information was collected from the medical record on age, breed and sex.

Anaesthetic records were reviewed for each enrolled patient. Anaesthetic premedication drug, dose and route were recorded, along with anaesthetic induction agent and use of benzodiazepines for co-induction. Use of anaesthetic maintenance agent was recorded, with patients classed as maintained on TIVA or PIVA. Continuous rate infusions (CRIs) utilised alongside the maintenance agents were recorded along with their rates. Attached monitoring equipment and arterial catheter placement was recorded for each patient. Administered fluid and antibiotic therapy was also recorded.

Animals were divided into two groups according to the maintenance anaesthetic agent used, with Group PIVA consisting of patients maintained on inhalational volatile anaesthetic agents alongside PIVA, and Group TIVA maintained on propofol infusion alongside other infusions. End-tidal sevoflurane or isoflurane concentrations were converted to minimum alveolar concentration (MAC) multiples using reference values of 1.27% for isoflurane (28) and 2.36% for sevoflurane (29). Infusion rate of propofol was converted to minimum infusion rate (MIR) multiples using reference value of 0.51 ± 0.08 mg kg ^−1^ min ^−1^ (30). The PIVA population was further divided to patients receiving lidocaine CRIs and those not receiving a lidocaine infusion for the purposes of analysing incidence of arrhythmias. Cardiovascular parameters from reviewed anaesthetic records were recorded at 10-min intervals during each anaesthetic with recorded numbers obtained by averaging two consecutive readings. This aimed to minimise the inaccuracy resulting from non-numerical values on anaesthetic records. Incidences of complications were identified, and the administration of any other pharmacological interventions was noted. Anaesthetic complications were defined as: tachycardia (> 160 beats minute^−1^), bradycardia [< 50 beats minute^−1^, presence of arrhythmias (yes/no)], hypotension [mean arterial pressures (MAP) < 60 mmHg or systolic arterial pressures (SAP) < 90 mmHg and hypertension (MAP > 100 mmHg or SAP > 160 mmHg)]. Further information was recorded on type of arrhythmias where data was available. Presence of each complication was assessed for each time interval in a yes/no approach enabling duration of complications to also be assessed. Incidence of arrhythmias were analysed at five-minute intervals to account for transient changes.

Interventions were classified further as administration of lidocaine bolus, fluid bolus (compound sodium lactate, CSL), infusions for blood pressure support or anticholinergic. Administered infusions for blood pressure support were phenylephrine, dopamine and dobutamine. Administered anticholinergics were atropine and glycopyrrolate. Surgical time was also recorded.

### Statistical analysis

Data was analysed using GraphPad Prism version 10.3.1 (GraphPad Software Inc., CA, United States). A *p* value of <0.05 was considered statistically significant.

The Shapiro–Wilk test was used to assess demographic data, incidence of hypotension, duration of hypotension and duration of anaesthesia for normality. Group demographic data, duration of hypotension and duration of anaesthesia were found to be non-normally distributed, while incidence of hypotension was normally distributed. Data on heart rate, SAP, MAP and DAP were found to be normally distributed by the D’Agostino and Pearson test. Group demographic data was compared using a Mann–Whitney test, and prevalence of brachycephalic breeds in each group was assessed using the Fishers Exact test. An unpaired Welch t-test was used to compare heart rate, SAP, MAP and DAP. SAP, DAP and MAP was also compared between maintenance with isoflurane and sevoflurane using an unpaired *t*-test.

Incidence of hypotension was assessed using the Fisher’s exact test. Relative risk was calculated for incidence of hypotension between treatment groups. Duration of hypotension was analysed using the Mann–Whitney test. The Fisher’s exact test was used to analyse incidence of bradycardia and tachycardia, administration of lidocaine boluses, fluid boluses, anticholinergics and vasopressors. Use of a lidocaine CRI, fentanyl CRI, combined lidocaine-fentanyl CRIs and benzodiazepine co-induction were also compared between groups using the Fisher’s exact test. Duration of anaesthesia was compared using a Mann–Whitney test.

Incidence of arrhythmias was assessed as three separate groups (TIVA, inhalational with lidocaine and fentanyl, and inhalational without lidocaine) and was analysed using the Kruskal-Wallis test.

## Results

In total, 44 dogs were analysed. 13 of these were maintained on TIVA and 31 dogs were maintained on PIVA anaesthesia with either isoflurane (*n* = 8) or sevoflurane (*n* = 23). Demographic data are displayed in Table 1. No significant difference was found in age (*p* = 0.953) or bodyweight (*p* = 0.063) between groups. The most represented breed was the French Bulldog, along with crossed/mixed breed dogs. Breed distribution is displayed in Table 2. The number of French bulldogs in each group was not statistically different (*p* = 0.362). No significant duration was found in duration of anaesthesia between groups (*p =* 0.259).

**Table 1:** Demographic data of study groups.

	PIVA group (*n* = 31)	TIVA group (*n* = 13)
Gender	Male *n* = 20, female *n* = 9	Male *n* = 5, female *n* = 8
Median and range of age (months)	12 (4–81)	11 (6–48)
Mean bodyweight ± standard deviation (kg)	11.8 ± 6.8	18.1 ± 9.6

**Table 2:** Breed data of study groups.

Breed	PIVA group (*n* = 31)	TIVA group (*n* = 13)
French Bulldog	10	3
Cross/mixed breed	3	3
Cocker Spaniel	3	0
Labrador	2	1
Greyhound	2	0
Whippet	0	2
Miniature Schnauzer	2	0
Other (breeds where *n* = 1)	9	4

### Anaesthetic protocol

Premedication drugs included methadone (Synthadon 10 mg mL^−1^, Animalcare, United Kingdom), pethidine (pethidine hydrochloride 50 mg mL^−1^, MercuryPharma, UK) and morphine (morphine sulphate, 10 mg mL^−1^ Hameln Pharma Ltd., United Kingdom). Premedication agents and induction agents used in each group are described in Tables 3, 4, respectively. Anaesthetic induction drugs included propofol (PropoFlo 10 mg mL^−1^, Zoetis, United States), alfaxalone (Alfaxan Multidose 10 mg mL^−1^, Dechra, United Kingdom) and etomidate (Hypnomidate, Janssen-Cilag Ltd., UK). Co-induction with midazolam (Midazolam 5 mg mL^-1,^ Hameln Pharma, UK) was included in many patients, including all dogs induced with etomidate (see Table 4). No statistical differences were seen between groups in the number of animals receiving a benzodiazepine co-induction (*p* = 0.0874).

**Table 3:** Premedication drugs used in each study group.

Premedication used	PIVA group (*n* = 31)	TIVA group (*n* = 13)
Methadone (0.2 mg/kg IV)	25	13
Pethidine (4-5 mg/kg IM)	5	0
Morphine (0.3 mg/kg IM)	1	0

**Table 4:** Induction agent data of study groups.

Induction agent	PIVA group (*n* = 31)	TIVA group (*n* = 13)
Propofol	7	8
Alfaxalone	2	0
Etomidate + midazolam (0.2–0.4 mg/kg)	3	0
Propofol + midazolam (0.2–0.5 mg/kg)	15	5
Alfaxalone + midazolam (0.2–0.4 mg/kg)	4	0

All patients were intubated with endotracheal tubes cuffed until no leak was detected and maintained on oxygen, with or without inhalant anaesthetic agent. Patients were connected to a multiparameter anaesthetic monitoring machine (Carescape Monitor B650, GE Healthcare, Finland). An arterial catheter was placed in the metatarsal artery in all dogs (22–25 gauge, Jelco IV catheters, Animalcare). Monitoring was performed at 5 -min intervals and included heart rate, respiratory rate, invasive blood pressure, end-tidal carbon dioxide partial pressure, end-tidal inhalant agent monitoring (where appropriate), pulse oximetry and constant rate infusion (CRI) rates in all cases. Capnography and electrocardiographic monitoring were used in all cases. Mechanical ventilation was utilised in 26/31 patients maintained on inhalant anaesthesia and 12/13 patients maintained on TIVA. Temperature was monitored at varying intervals in all cases. All included valvuloplasties were performed using a jugular venous approach.

Inhalant maintenance was carried out with either isoflurane (Iso-Flo, Abbott Laboratories UK Ltd., United Kingdom) or sevoflurane (SevoFlo, Zoetis, Belgium). Of the PIVA group, 11/31 were maintained on isoflurane and 23/31 were maintained on sevoflurane. Mean maintenance rates of sevoflurane was 1.59 ± 0.40% (0.69 ± 0.17 MAC) and for isoflurane was 1.02 ± 0.28% (0.77 ± 0.12 MAC). While adjustment of end tidal volatile agent was at the discretion of the anaesthetist, all patients were maintained below defined MAC values for the agent throughout the study. When TIVA was utilised, this was performed using propofol (Propoflo-Lipuro 10 mg mL^−1^, B. Braun, Germany) alongside additional CRIs as described below. In the TIVA group, the mean propofol maintenance rate was 0.25 ± 0.09 mg kg minute^−1^ (0.49 ± 0.17 MIR).

Additional CRIs used during the anaesthetic were recorded. These included fentanyl (Fentadon 50 mcg mL^−1^, Dechra, United Kingdom), lidocaine (Lidocaine hydrochloride 2%; B. Braun Medical Ltd., Ireland) and both fentanyl and lidocaine in combination. Additional CRIs utilised are described in Table 5. No statistical differences were seen between groups in the number of animals receiving a fentanyl CRI (*p* = 0.0569), or combined use of lidocaine and fentanyl CRIs (*p* = 0.0610). CRIs used alongside the inhalant anaesthetic in the PIVA group included fentanyl (*n* = 3/31, 0–0.3 mcg kg min^−1^, mean rate 0.1 mcg kg min^−1^) and lidocaine (*n* = 2/31, 0–80 mcg kg min^−1^, mean rate 29.2 mcg kg min^−1^). A combination of lidocaine and fentanyl was used in 26 patients receiving PIVA (*n* = 26/31). In the TIVA group, additional CRIs used included fentanyl (*n* = 6/13, 0–0.3 mcg kg min^−1^, mean rate 0.1mcg kg min^−1^) and lidocaine (*n* = 3/13, 0–80 mcg kg min^−1^, mean rate 23.5 mcg kg min^−1^). Lidocaine and fentanyl were combined in 4 patients (*n* = 4/13).

**Table 5:** Additional CRIs administered to each study group.

Additional CRIs administered	PIVA group (*n* = 31)	TIVA group (*n* = 13)
Fentanyl (alone)	3	6
Lidocaine (alone)	2	3
Fentanyl and lidocaine	26	4

All patients received peri-operative cefuroxime (Zinacef, Glaxo-SmithKline Ltd., UK) at 90 min intervals throughout the procedure and Hartmann’s intravenous fluid solution (Vetivex 11, Dechra, UK) at 3–5 mL kg hour ^−1^. Acepromazine was administered before recovery in *n* = 7/31 groups in the PIVA group at 2.5–5 mcg kg^−1^, and *n* = 2/13 patients in the TIVA group at 5 mcg kg^−1^ (Acecare 2 mg mL^−1^, Ecuphar, UK). Non-steroidal anti-inflammatory drugs (meloxicam, Metacam 5 mg/mL injectable solution, Boehringer Ingelheim, France) were administered to *n* = 2 patients in each group following recovery.

### Blood pressure

**Figure 1:**
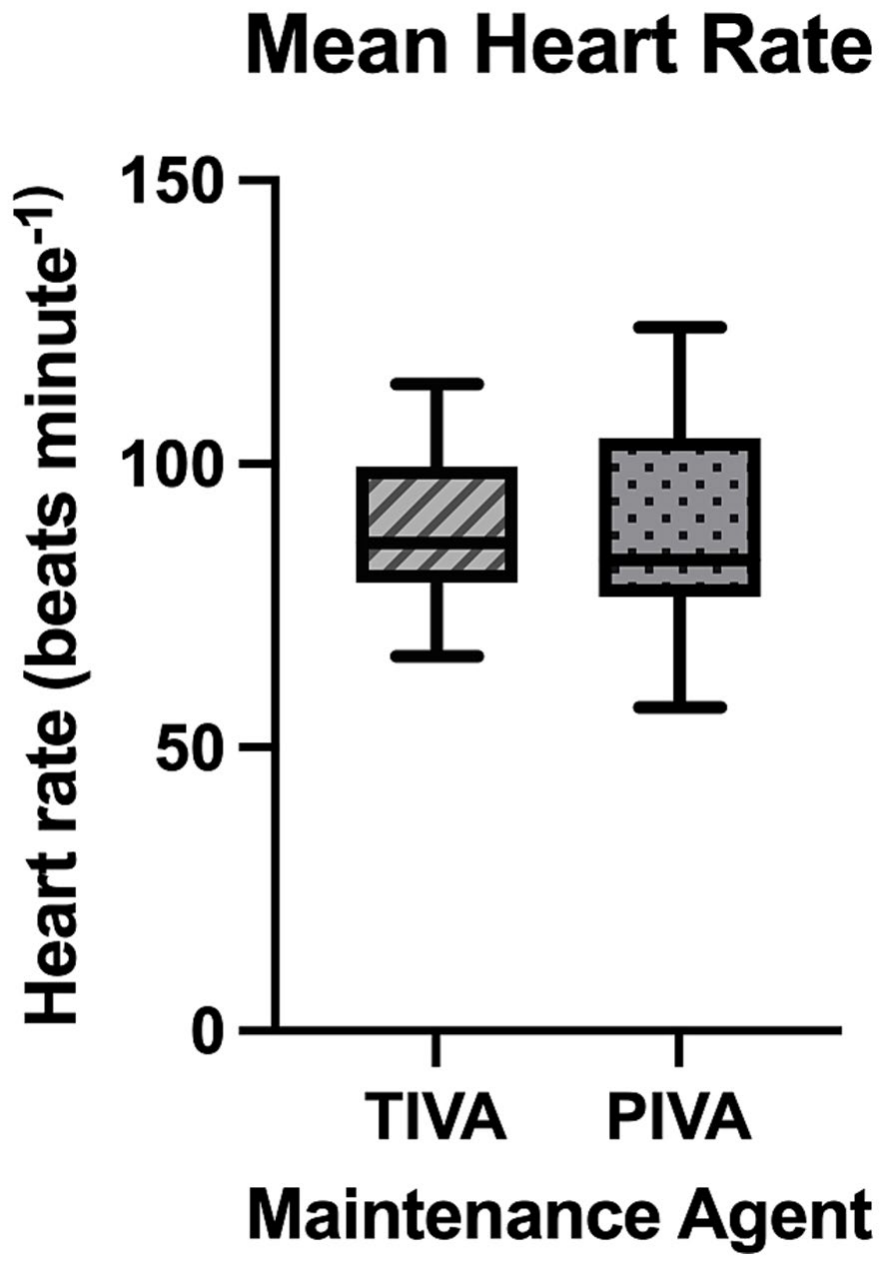
A box and whisker plot representing total duration of hypotension seen with each maintenance agent.

Average SAP was found to be significantly higher in patients maintained on TIVA (106 ± 18 mmHg) compared to those maintained on PIVA (96 ± 15 mmHg; *p* = 0.0397). Average MAP and DAP were also significantly higher in patients maintained on propofol (73 ± 8 mmHg, 59 ± 8 mmHg) versus those on PIVA (65 ± 9 mmHg, 52 ± 8 mmHg) (*p* = 0.008, 0.016 respectively). Incidence of hypotension was significantly higher in patients with PIVA (78.79%) compared to TIVA (40%), OR 0.18, 95% CI: 0.052–0.66 (*p* = 0.019). The relative risk of developing hypotension in the PIVA group was 1.97. Total duration of hypotension was also significantly lower in patients on TIVA (median 40, IQR 10–65.5 min) compared to those on PIVA (median 65, IQR 17.5–110 min) (*p* = 0.003; see Figure 1). Incidence of hypertension was not significantly different between groups (*p* = 0.204), with *n* = 2/13 patients on TIVA and *n* = 1/31 patients on PIVA experiencing hypertension. No incidence of hypertension was prolonged beyond a single reading. No significant difference was found between isoflurane and sevoflurane maintenance in SAP, MAP and DAP (*p* = 0.070, *p* = 0.08, *p* = 0.68 respectively).

### Heart rate

**Figure 2:**
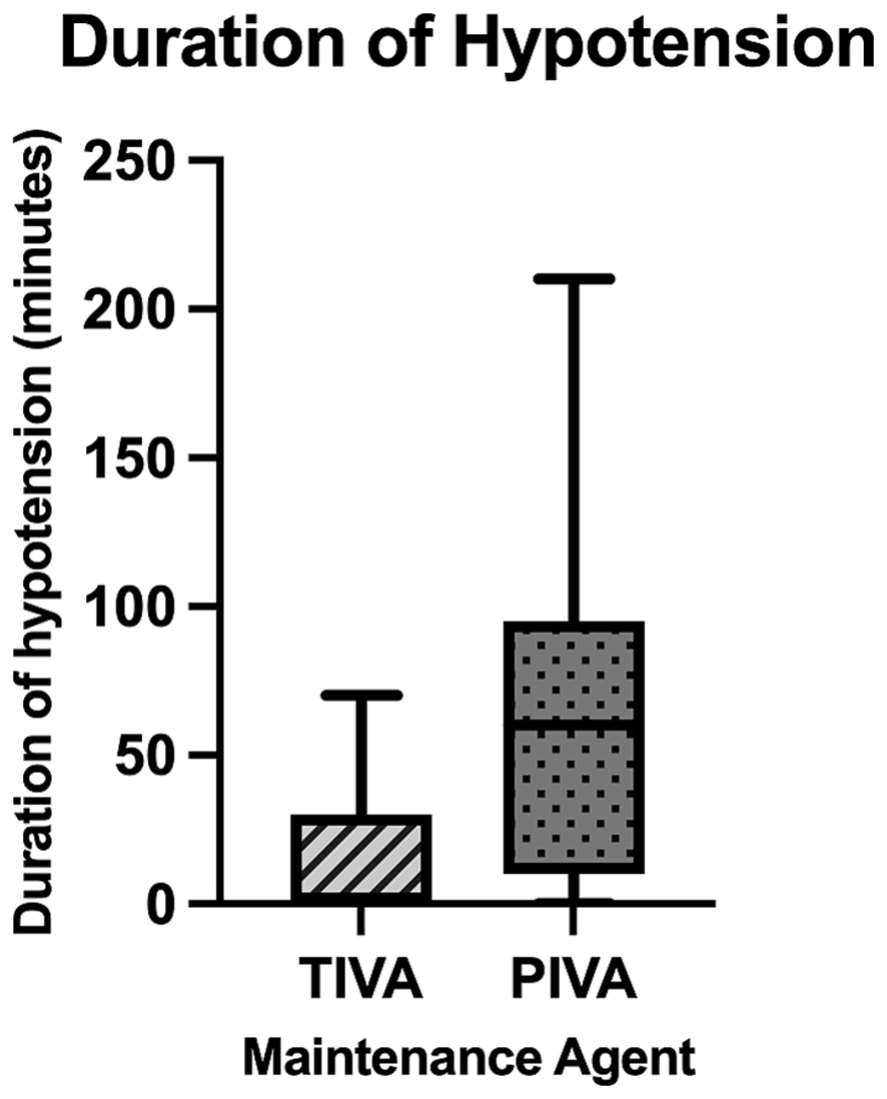
A box and whisker plot representing mean heart rate of patients maintained on each maintenance agent.

No significant difference was found between heart rates between dogs maintained on propofol (88 ± 14 beats minute^−1^) and those maintained with PIVA (88 ± 18 beats minute ^−1^) (*p* = 0.953; see Figure 2). No statistically significant difference in occurrence of tachycardia was seen (*p* > 0.999), with tachycardia seen in n = 9/31 dogs on PIVA maintenance and *n* = 4/13 dogs on TIVA maintenance. No statistically significant difference in occurrence of bradycardia was seen (*p =* 0.570), with *n* = 2/31 dogs on PIVA and *n* = 2/13 dogs on TIVA experiencing bradycardia.

### Arrhythmias

Incidence of reported arrhythmias was not statistically different between groups (*p* = 0.488). The origin of arrhythmias was not mentioned in the majority of the anaesthetic records. From the ones specified, ventricular arrhythmias were the most reported (reported in 12/48 cases). Other recorded arrhythmias included right bundle branch block (1 case), atrioventricular block (1 case, type not specified) and tachyarrhythmia (1 case, type not specified).

### Interventions

There was no significant difference between the two groups regarding the number of lidocaine boluses given (*p* = 0.295) and of fluid boluses (*p* = > 0.999). Use of infusions for blood pressure support was also not significantly different between groups (*p* = > 0.999), with 15.4% of patients on TIVA (*n* = 2/13) and 19.4% of patients on PIVA (*n* = 6/31) receiving vasopressors. Vasopressors used included dobutamine (*n* = 1 patient on PIVA), dopamine (*n* = 2 patients on TIVA, *n* = 4 patients on PIVA) and phenylephrine (*n* = 1 patient on PIVA). Use of anticholinergics was also not significantly different between groups (*p* = 0.746) with 26.7% of patients on TIVA and 33.3% of patients on PIVA receiving them. Anticholinergics administered included glycopyrrolate (*n* = 4/31 dogs on PIVA and *n* = 3/13 dogs on TIVA) and atropine (*n* = 1/31 dogs on PIVA, no dogs on TIVA). Two patients in the PIVA group were administered atracurium, no patients maintained on TIVA were given atracurium.

Use of a lidocaine CRI was significantly higher in patients maintained on with PIVA (87.9%) than those maintained on propofol (53.3%), (*p* = 0.022).

## Discussion

The main finding of this study was that dogs maintained on TIVA for pulmonic balloon valvuloplasty had a lower incidence and duration of hypotension compared to those maintained on PIVA.

One explanation for the increased incidence and duration of hypotension in the PIVA group could be the vasodilatory effects of isoflurane (10). Our finding are in agreement with previous studies that showed improved mean arterial pressures when maintaining anaesthesia in dogs with a propofol TIVA compared to isoflurane inhalational agents (10, 25, 26). Ramos et al. (7) observed hypotension in 48.7% of dogs anaesthetised using PIVA for balloon valvuloplasty. While the procedure itself likely contributed to some cases, 28.2% of the dogs were hypotensive before the intervention, implicating the anaesthetic maintenance agent as a possible cause. Intraoperative hypotension is a common peri anaesthetic complication (31). It is associated with significant postoperative complications including acute kidney injury, myocardial injury and death, with human research showing injury from durations of arterial hypotension as brief as 1–5 min (32). In the present study, maintenance of higher arterial blood pressures overall, alongside a lower incidence and duration of hypotension, supports maintenance of better cardiovascular stability in these patients. The relative risk (RR) of developing hypotension in the PIVA group was 1.97, indicating that patients in this group were nearly twice as likely to experience hypotension compared to the propofol group. It is worth noting that while a statistically significant difference in average systolic, mean and diastolic arterial blood pressure values was found in this population, the magnitude of difference in this study is unlikely to be of clinical significance.

The incidence of arrhythmias was not found to be significantly different between patients maintained on TIVA or PIVA in this study. Due to the limitations of retrospective data, the type of arrhythmia seen was not always specified, and the stage of the interventional procedure was not always noted when arrhythmias were recorded. While arrhythmias can occur at many stages of balloon valvuloplasty, manipulation of equipment in the right ventricular outflow tract commonly results in ventricular arrhythmias (5) regardless of anaesthetic protocol or intervention. Due to the higher rate of use of a prophylactic lidocaine CRI in patients on PIVA anaesthesia proving a potentially confounding factor, patients receiving inhalant anaesthesia were further subdivided to those receiving a lidocaine CRI and those that did not, with no statistically significant difference in incidence of arrhythmias found between groups. A retrospective study by Phillips et al. (33) found no significant difference in incidence or malignancy of arrhythmias during balloon valvuloplasty in dogs pre-treated with a lidocaine bolus and CRI, theorising that arrhythmias caused by mechanical stimulation of the myocardium are resistant to management with lidocaine. This suggests that the increased use of a lidocaine CRI in the patients receiving inhalant anaesthetics is unlikely to have influenced results. Further prospective studies with standardised recording protocols are warranted to further explore if any differences in incidence or type of arrhythmias are seen between PIVA and TIVA maintenance.

There was no significant difference in heart rate between the two maintenance agents, or incidence of anticholinergic administration. This contrasts with Keegan & Greene’s (26) paper where propofol was associated with maintenance of a higher heart rate. As both cardiac output and systemic vascular resistance play a crucial role in maintaining blood pressures in the anaesthetised patient, the lack of difference in heart rate between groups may suggest that maintaining systemic vascular resistance played a major role in the higher blood pressures documented by this study. Pre-operative administration of atenolol was not assessed in this population, which has been associated with a lower mean heart rate during the procedure (34). Dexmedetomidine CRIs were not utilised in any of the assessed population, which has been associated with reduced vasopressor and anticholinergic requirement (20).

Our results support the secondary null hypothesis that there would be no significant difference in pharmacological interventions to maintain cardiovascular stability between patients maintained on TIVA or PIVA anaesthesia. This is an interesting finding, as it would be expected the higher incidence of hypotension to necessitate a greater number of interventions to maintain haemodynamic stability. However, this can likely be explained by the small sample size and the retrospective nature of the study meaning that interventions were not standardised. Additionally, fluid boluses can be administered preventatively prior to balloon inflation in some cases, which may have resulted in similar results between groups as the preventative nature may not be recorded.

### Limitations

This study has several limitations. The retrospective nature of this study means that administered CRIs, CRI rates, end tidal volatile agents and ventilation parameters were not standardised in the study population, all of which may have influenced cardiovascular parameters and depth of anaesthesia in this study. However, this represents the clinical reality of treating these cases and managing inter-patient variability. Temporal changes in anaesthetic management and cardiologist technique may have also influenced the study findings, as cases were included over a nine-year period. This means that changes in other aspects of clinical work may have occurred during this period, adding additional reasons for the reduced rate of hypotension. Variation between anaesthetists may have influenced results, however all cases were supervised by an anaesthesia diplomat or residency trained clinician. Increased usage of TIVA over time due to environmental implications and increased clinician experience may also be a confounding factor, as a greater proportion of the cases maintained on TIVA were performed in the later period of data collection. All protocols were at the discretion of the treating anaesthetist thus varied between patients. Lack of access to patients to evaluate anaesthetic depth due to patient draping, and lack of recording of patient depth assessments, could result in a source of error. Additionally, the retrospective nature of this study resulted in unequal group sizes. While appropriate statistical analyses were chosen for this, the findings should be interpreted with consideration of this imbalance.

Miller et al. (31) found brachycephalic breeds to have greater odds of hypotension under anaesthesia, however the number of French bulldogs was not statistically significant between groups. Additionally, while hypoventilation is a recognised complication of propofol TIVA (10), this could not be assessed by this study due to the varying use of mechanical ventilation. Pulmonic valve pressure gradient was not consistently measured or assessed in this study and the extent of the effect of inhalant agents and propofol on this gradient in animals is not known. Future studies to investigate the impact of maintenance agent on pulmonic valve pressure gradient would be of interest to investigate this relationship.

Target-controlled infusion (TCI) was not used in this study, with CRI rates instead determined at the anaesthetist’s discretion. Use of TCI of propofol in dogs has been demonstrated to result in less fluctuations in predicted plasma concentrations and a lower risk of hypotension when compared to CRI (35). This suggests that the beneficial effects of propofol TIVA on blood pressure shown by this study may be greater if a TCI pump was used instead of anaesthetist-controlled TIVA. Future studies comparing the use of a TCI pump for TIVA during balloon valvuloplasty would be of interest to confirm this suggestion.

## Conclusion

The findings of this retrospective study suggest that in the population studied here, maintenance of anaesthesia with propofol TIVA resulted in higher blood pressure and a lower incidence and total duration of hypotension than PIVA anaesthesia in dogs undergoing balloon valvuloplasty for treatment of pulmonic stenosis. However due to the diverse anaesthetic protocols used within this retrospective study, further prospective studies are required to confirm the results of this paper.

## Data Availability

The raw data supporting the conclusions of this article will be made available by the authors, without undue reservation.
